# The Antioxidant Properties and Inhibitory Effects on HepG2 Cells of Chicory Cultivated Using Three Different Kinds of Fertilizers in the Absence and Presence of Pesticides

**DOI:** 10.3390/molecules200712061

**Published:** 2015-07-01

**Authors:** Jin-Seon Yook, Mina Kim, Pichiah BalasubramanianTirupathi Pichiah, Su-Jin Jung, Soo-Wan Chae, Youn-Soo Cha

**Affiliations:** 1 Department of Food Science and Human Nutrition, Chonbuk National University, 664-14 Duckjin-dong, Jeonju, Jeonbuk 561-756, Korea; E-Mails: y1126sun@hanmail.net (J.-S.Y.); als@jbnu.ac.kr (M.K.); tirupathipichiah@gmail.com (P.B.T.P.); 2 Clinical Trial Center for Functional Foods, Chonbuk National University Hospital, 20, Geonjiro, Deokjin-gu, Jeonju, Jeonbuk 561-712, Korea; E-Mails: sjjeong@jbctc.org (S.-J.J.); soowan@jbnu.ac.kr (S.-W.C.)

**Keywords:** chicory, anti-oxidants, phenolic compounds, fertilizer, pesticide

## Abstract

The objective of this study was to explore the antioxidant levels and anticancer properties of chicory cultivated using three different kinds of fertilizers (*i.e.*, developed, organic, and chemical) in the presence and absence of pesticides. Phenolic phytochemicals, including total polyphenols and flavonoids, and antioxidant activities, including reducing power, ABTS^+^ and DPPH radical scavenging activity, were analyzed using several antioxidant assays. HepG2 cell viability was analyzed using the MTT assay. The antioxidant properties of chicory were found to increase when cultivated with chemical fertilizer in the absence of pesticides. On the other hand, antioxidant capacity was higher in chicory cultivated with eco-developed fertilizer even in the presence of pesticides. Chicory grown using eco-developed or organic fertilizer was more effective in suppressing the proliferation of HepG2 cells when compared to chicory grown with chemical fertilizer. This effect was time dependent, regardless of treatment with or without pesticides. In conclusion, the antioxidant activity of chicory were affected by the presence or absence of pesticides. However, developed and organic fertilizers showed a strong anti-proliferative effect against HepG2 cells, regardless of the presence or absence of pesticides.

## 1. Introduction

The growing world economy has improved the clinical diagnosis and treatment of many chronic diseases, but the incidence of diseases like cancer has increased rapidly and is remains the leading cause of mortality worldwide [1,2,3].

Moreover, medical costs for such chronic diseases are very high [4]. Therefore, to cope with the increased incidence of chronic diseases, it is necessary to take preventive measures by changing one’s lifestyle, including food habits. The diet plays a crucial role in regulating the expression of many genes and therefore it plays a vital role in health and diseases [5,6]. Hence, intake of the right kind of diet can help avert many chronic diseases [7,8]. Phytochemicals in fruits and vegetables have been reported to induce changes in the expression of many genes that are involved in health and diseases [9]. One of the well-known sources of phytochemicals is vegetables, which are high in antioxidants and play an important role in averting chronic diseases like cancer and cardiovascular disease [10,11].

In the past, the agriculture industry was mainly concerned with increasing the production of crops. Therefore, different kinds of chemical fertilizers and pesticides were utilized [12,13]. Extensive usage of such chemicals greatly improves the productivity of food crops, but with compromised nutritional status. In 2011, the magazine Scientific American reported that the nutrients of carrots in the 1970s were higher than in carrots today, because of the depletion of nutritional ingredients in the soil resulting from extensive use of fertilizers and pesticides, greenhouse cultivation, and genetically modified species of crops has led to the production of fruits and vegetables with lower nutritional value [14].

Vegetables with higher nutritional value have greater demand in the consumer market. However, nutritional components, such as the content of antioxidants and other phytochemicals including total polyphenols and flavonoids, of vegetables depends on several factors such as the type of fertilizer [12,15,16] and pesticide [17], maturity [18], and planting period [19].

Among the different vegetables, we focused on chicory (*Cichorium intybus* L.) for several reasons. Chicory is a chrysanthemum family plant with a wealth of health benefits. Being rich in phenolic compounds (e.g., flavonoids and polyphenols), it has antioxidative and anticancer effects [20,21,22]. The highest levels of phenolic compounds and highest activities of antioxidants are found in the leaves of chicory than other parts [23]. It is enjoyed with different meals as a salad. To improve the beneficial properties of chicory, interventions are needed. Instead of chemical pesticides, the use of organic fertilizers like compost and vermicompost has become a welcomed practice recently. Organic fertilizers have received more attention worldwide due to the growing concern over environmental safety and human health issues. On the other hand, it remains controversial as to whether crops cultivated by organic agriculture are healthier than those cultivated with chemical fertilizers and pesticides [1,24,25,26,27]. There are no report available showing changes in the phytochemical profile of chicory cultivated with organic versus chemical fertilizer. Furthermore, no reports have demonstrated how changes in cultivation conditions affect chicory’s antioxidant levels. Therefore, we cultivated chicory using organic or chemical fertilizer with or without pesticide to find out whether the pesticide and/or the type of fertilizer affect the nutritional value and health benefits of chicory.

## 2. Results and Discussion

### 2.1. Components of Chicory

Moisture was significantly higher in the non-pesticide groups compared to the pesticide-treated groups (Table 1), and among the non-pesticide group, treatment with chemical fertilizer was beneficial for raising the moisture content in the chicory plants. As revealed by two-way ANOVA, the moisture content was affected by the fertilizer treatment as well as the pesticide availability (Table 2) indicating an interaction between pesticide and fertilizer. The amount of ash content varied, depending on the pesticide availability and fertilizer treatment; however, no interaction between pesticide and fertilizer was observed (Table 2). Chicory plants treated with chemical as well as eco-developed fertilizer showed significantly higher amount of ash, regardless of pesticide availability. Shier *et al.* [28] reported that conventional farming conditions improve moisture contents compared to organic farming. Shier’s and our results suggests that pesticide and fertilizer have an impact on ash and moisture availability, and moreover among the three different fertilizers tested, chemical fertilizer enhances the ash and moisture contents compared to organic (eco and org) fertilizers.

**Table 1:** Moisture and ash composition of chicory.

Groups		Nutrients (g/100 g)	
		Moisture	Ash
NP	eco	6.51 ± 0.03 ^b^	16.20 ± 0.31 ^a,b^
	org	6.97 ± 0.22 ^b^	15.63 ± 0.09 ^b^
	che	7.92 ± 0.13 ^a^	16.70 ± 0.19 ^a^
LP	eco	5.43 ± 0.12 ns	16.77 ± 0.00 ^x^
	org	5.76 ± 0.07	16.26 ± 0.09 ^y^
	che	5.70 ± 0.16	16.77 ± 0.20 ^x^

All values are given as mean ± standard deviation. Values with different letters denote statistical difference (*p* < 0.05). Letters a, b are used to denote comparisons within NP groups; letters x, y are used to denote comparisons within LP group. ns: non-significant; NP: non-pesticide group; LP: low-pesticide group; eco: eco-developed fertilizer; org: organic fertilizer; che: chemical fertilizer.

**Table 2:** ANOVA of Means Square for moisture, ash, phytochemical compound in chicory.

	Moisture	Ash	Total Polyphenols	Total Flavonoids	Reducing Power	DPPH Radical Scavenging Activity	ABTS^+^ Radical Scavenging Activity
Pesticide (P)	6.78 **	0.54 *	842.69 *	112.50 ns	0.051 *	141.64 **	1.79 ns
Fertilizer (F)	0.70 **	0.66 *	409.59 *	26.39 ns	0.030 *	147.38 **	0.32 ns
P × F	0.39 *	0.10 ns	1361.58 **	3912.5 **	0.129 **	176.26 **	0.2 ns

ns: non-significant, * *p* < 0.01, ** *p* < 0.001.

### 2.2. Antioxidant Content (Total Polyphenols and Flavonoids) of Chicory

**Figure 1:**
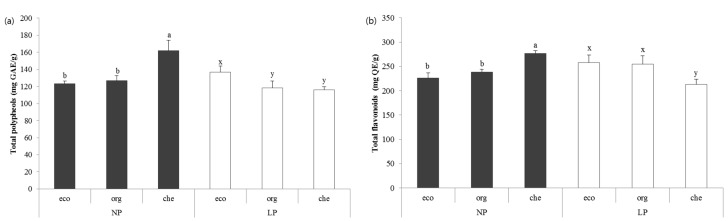
(**a**) Total polyphenol content of chicory; (**b**) Total flavonoid content of chicory. All values are expressed as means ± standard deviation. Values with different letters denotes statistical difference (*p* < 0.05). Letters a, b are used to denote comparisons within NP groups; letters x, y are used to denote comparisons within LP group. NP: non-pesticide group, LP: low-pesticide group. eco: eco-developed fertilizer, org: organic ferilizer, che: chemical fertilizer.

Pesticide and fertilizer had a direct effect as well as interaction effect on the amount of polyphenols in chicory; indicating that the total polyphenols content depends on the availability of pesticide and the type of fertilizer used. The NP-che group (162.14 mg GAE/g) showed the highest amount of polyphenols, followed by NP-org (127.05 mg GAE/g) and NP-eco (123.19 mg GAE/g). While in the pesticide group, the total polyphenol content was highest in LP-eco (136.88 mg GAE/g), followed by LP-org (118.28 mg GAE/g) and LP-che (116.18 mg GAE/g) (Figure 1a). Under pesticide-free conditions the total flavonoids content in chicory cultivated with chemical fertilizer [NP-che (276.67 mg QE/g)] was higher compared to eco-developed and organic fertilizer [NP-eco (226.67 mg QE/g) and NP-org (238.33 mg QE/g)]. It was observed that in presence of pesticide, the eco-developed fertilizer yields highest level of flavonoids [LP-eco (258.33 mg QE/g)] (Figure 1b). Reactive oxygen species (ROS) are chemically reactive molecules that damages organs by attacking lipids, proteins, and even DNA under conditions of oxidative stress [29]. Consumption of leafy vegetables containing high antioxidant averts many chronic diseases by acting as a scavengers and reducing agents that remove free radicals while being oxidized itself and thereby protecting the cells [30,31,32,33]. Thus vegetable with phenolic compounds advocates its quality. Therefore there is always a quest for devising methods for improving the phenolic content in the vegetables. So far studies for improving phenolic profiles in vegetables using different fertilizer treatment led to different results.

Some studies have reported that organic fertilizers had a better impact on plant phenolic composition than chemical fertilizers [34], while others showed no significant difference in total phenolic content between organic and chemical fertilizer treated groups [15]. However, some studies have suggested that chemical fertilizers improve the phenolic content [21,28]. It was also noticed that pesticide treatment affects the phenolic content of the plant [35]. As the phenolic compounds in the vegetable are considerably influenced by cultivar, season, and other environmental factors, therefore apart from the types of fertilizers, our results might be also influenced by the availability of pesticides.

### 2.3. Antioxidant Capacity of Chicory

**Figure 2:**
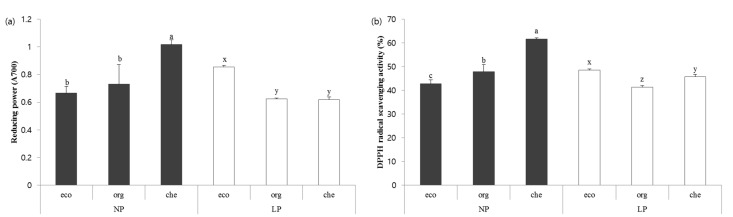
(**a**) Reducing power of chicory; (**b**) DPPH radical scavenging activity. All values are expressed as means ± standard deviation. Values with different letters denote statistical differences (*p* < 0.05). Letters a–c are used to denote comparisons within NP groups; letters x–z are used to denote comparisons within LP group. NP: non-pesticide group, LP: low-pesticide group. eco: eco-developed fertilizer, org: organic fertilizer, che: chemical fertilizer. Sample concentration: 1 mg∙mL^−1^.

We measured the reducing power and free radical quenching capacity of chicory by measuring the quenching of DPPH and ABTS^+^ radicals. The two-way ANOVA showed significant results, indicating that both the fertilizers and pesticide had a direct and an interaction effect (P × F) on the reducing power and DPPH radical scavenging activity. The reducing power of extracts from chicory grown in the absence of pesticide showed results conflicting to that of extracts from chicory grown in pesticide-treated soil (Figure 2a). In absence of pesticide, chemical fertilizer-treated group (NP-che) showed the highest reducing power. However, in the presence of pesticide, plants treated with eco-developed fertilizer (LP-eco) gave the highest reducing power. Moreover, this matched the levels of total polyphenols and flavonoids.

Higher DPPH radical scavenging activity (Figure 2b) was observed in the chicory cultivated with chemical fertilizer without pesticide (NP-che: 61.63%). Extracts of chicory grown in pesticide-treated soil with eco-developed fertilizer followed by chemical fertilizer (LP-eco: 48.47% and LP-che: 51.67%) had higher DPPH radical scavenging activity compared to organic fertilizer. We thus observed that the DPPH radical scavenging activity was influenced by pesticide and/or fertilizer. Even though there was no significant direct or interaction effect, it was worth noting that the ABTS^+^ radical scavenging activity of chicory extracts was similar to that of Trolox which was used as a positive control (Table 3).

Some of the phenolics in chicory may act as antioxidants, and the antioxidant capacity of these phenolics is due to the hydrogen donating ability of hydroxyl groups in their structure [32]. These hydroxyl groups reduce radicals, therefore higher phenolics lead to higher antioxidant capacity [36]. Our data agree with the Velioglu *et al.* study [37] in which they reported that total phenolic and antioxidant activities are strongly associated each other. Accordingly, higher content of antioxidants was observed in chicory cultivated with chemical fertilizer in the absence of pesticide or grown using eco-developed fertilizer in presence of pesticide promoted raise in the antioxidants such as total polyphenols and flavonoids leading to higher antioxidant activities in these groups.

**Table 3:** ABTS^+^ radical scavenging activity, total carotenoids and vitamins in chicory.

Groups		ABTS^+^ Radical Scavenging Activity (%)	Total Carotenoids (mg/100 g)	Vitamin C (mg/100 g)	Folic Acid (mg/100 g)
NP	eco	98.84 ± 0.20 ns	120.37 ± 5.46 ^a^	98.34 ± 12.42 ns	110.03 ± 15.07 ns
	org	98.69 ± 0.23	114.97 ± 12.0 ^b^	98.15 ± 3.76	103.23 ± 6.99
	che	98.46 ± 0.07	84.49 ± 6.00 ^c^	96.89 ± 1.72	98.70 ± 24.03
LP	eco	98.37 ± 0.34 ns	108.02 ± 2.18 ^x^	664.25 ± 25.92 ns	119.06 ± 15.88 ns
	org	97.64 ± 1.56	90.28 ± 0.00 ^y^	666.46 ± 2.23	105.51 ± 11.87
	che	98.08 ± 0.33	81.79 ± 0.00 ^z^	634.10 ± 42.76	79.52 ± 18.52

All values are given as means ± standard deviation. Values with different letters denote statistical difference (*p* < 0.05). Letters a–c are used to denote comparisons within NP groups; letters x–z are used to denote comparisons within LP group. ns: non-significant; NP: non-pesticide group; LP: low-pesticide group; eco: eco-developed fertilizer; org: organic fertilizer; che: chemical fertilizer. Sample concentration used in ABTS^+^ radical scavenging activity: 1 mg∙mL^−1^.

### 2.4. Total Carotenoids and Vitamins of Chicory

The total carotenoids and Vitamin C levels are shown in Table 3. The levels of total carotenoids were significantly higher in extracts of chicory grown with eco-developed fertilizer in the presence or absence of pesticide. The results for total carotenoids by two-way ANOVA showed significant effects of pesticide and fertilizer (Table 4) however no interaction between pesticide and fertilizer was observed for the outcome of total carotenoids, thus indicating that total carotenoid content differs depending on the addition of pesticide and the three different types of fertilizer but not on the interaction between pesticide and fertilizer. The levels of ascorbic acid were not changed by the types of fertilizer but were changed significantly with pesticide treatment (Table 4) as an about six-fold increase in vitamin C was observed in the pesticide group compared to the non-pesticide treated group. Plants synthesis vitamin C in response to chemical induced stress, one study showed that potato tubers treated with pesticide had higher level of vitamin C than control in order to cope the pesticide induced stress [38]. Therefore in this study also chicory plant cultivated in presence of pesticide to combat the pesticide induced stress, which was not observed as a result of fertilizer treatment. There was no significant difference observed in the folic acid levels among the groups; however, the folic acid level was higher in the eco-developed fertilizer group, irrespective of pesticide (Table 3) regardless of the presence or absence of pesticide. 

**Table 4:** ANOVA of means square for total carotenoids, vitamins, HepG2 cells viability, weight and length in chicory.

	Total Carotenoids	Vitamin C	Folic Acid	HepG2 Cells Viability	Weight	Length
Pesticide (P)	526.36 *	1396831.35 **	189767.55 **	118.76 ns	12012.50 **	174.22 **
Fertilizer (F)	985.39 *	532.98 ns	30.94 ns	769.34 **	938.29 ns	20.22 ns
P × F	121.51 ns	449.21 ns	983.45 ns	342.27 *	281.17 ns	0.89 ns

ns: non-significant, * *p* < 0.01, ** *p* < 0.001.

### 2.5. Weights and Length of Chicory

The weight and length of the chicory plant is shown in Table 5. There was no significant difference observed in the weight or length of chicory plant with respect to fertilizer treatments. However, it was noticed that, the growth of the plant was influenced by the availability of pesticide (Table 4) while no interaction between pesticide and fertilizer was observed. The size of the chicory plant without pesticide treatment was smaller, whereas the size of the chicory plant grown with pesticide was bigger.

Ascorbic acid plays an important role not only as an antioxidant, but also in growth and development. Rashida *et al.* [39] and Esteban *et al.* [40] reported that ascorbic acid significantly increased along with fruit growth and development regardless of cultivars. As we observed that pesticide influenced the Vitamin C level, therefore it could be possible that high level of vitamin C might triggered the growth of the plant in response of the chemical stress induced by the pesticide [38,41,42,43].

**Table 5:** Weight and length of chicory.

Groups		Weight (g)	Length (cm)
NP	eco	58.33 ± 22.68 ns	45.67 ± 4.93 ns
	org	70.67 ± 2.52	49.00 ± 3.00
	che	69.67 ± 7.77	69.67 ± 1.53
LP	eco	100.33 ± 0.12 ns	52.33 ± 2.52 ns
	org	138.00 ± 22.72	55.67 ± 1.15
	che	115.33 ± 6.03	52.00 ± 2.65

Three plants of chicory were randomly selected and measured weight and length. Two factors (weight and length) were non-significant in both NP and LP groups. NP: non-pesticide group; LP: low-pesticide group; eco: eco-developed fertilizer; org: organic fertilizer; che: chemical fertilizer; ns: non-significant.

### 2.6. Inhibitory Effect of Chicory on HepG2 Cells

**Figure 3:**
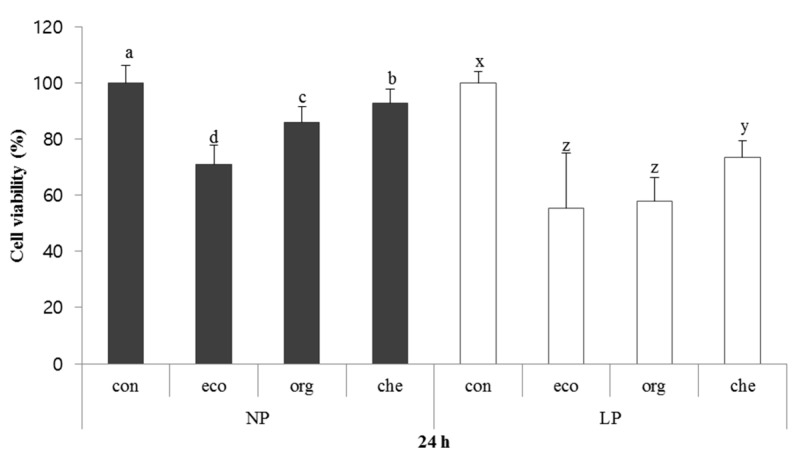
Viability of HepG2 cells cultured with different chicory extracts for 24 h. All values are expressed as means ± standard deviation. Values with different letters denote statistical difference (*p* < 0.05). Letters a–d are used to denote comparisons within NP groups; letter x–z is used to denote comparisons within LP group. NP: non-pesticide group, LP: low-pesticide group. eco: eco-developed fertilizer, org: organic fertilizer, che: chemical fertilizer. Sample concentration: 1 mg∙mL^−1^.

The human hepatocellular carcinoma (HepG2) inhibitory effect of chicory was investigated *in vitro* using HepG2 cells (Figure 3). The HepG2 cells were cultured for 24 h with extracts from chicory grown with different types of fertilizers in the presence or absence of pesticide. The direct effects of pesticide and fertilizer on HepG2 cells viability were significant, but no interaction was observed. The eco-developed fertilizer had an obvious inhibitory effect on HepG2 cells, both in the absence and presence of pesticide; however, the group with low pesticide and organic fertilizer also had a marked effect on the inhibition of HepG2 cell growth. In the absence of pesticide, the inhibition rate of NP-eco compared with the control was 28.91% while for LP-eco and LP- org it was 55.37% and 44.22% respectively.

These results indicate that the eco-developed fertilizer had a potent anti-proliferative effect against HepG2 cell. One of the reasons for the inhibitory effect could be due to content of carotenoids and vitamin C. It has been reported that carotenoids, a natural fat-soluble pigments that have isoprenoid polyenes, are effective against cancer development and act as chemopreventive agents [44]. Moreover, vitamin C is well known for its antioxidant activity, which is correlated with reduced risk of cancer development. However, the anticancer activity does not result for a single effect, but combined effects of several components found in vegetables and fruits [45]. Therefore, carotenoids and vitamin C could potentially inhibit cell growth by enhancing levels of antioxidants and the clearance of ROS in cancer cells.

## 3. Experimental Section

### 3.1. Chicory Cultivation

Chicory (*Cichorium intybus* L.) was cultivated for 2 months in a vinyl greenhouse at Jeongeup, Korea. The whole growth process is managed through the Good Agricultural Practices (GAP) program certified by the National Agricultural Products Quality Management Service (NAQS). The Good Agricultural Practices program aims to control agricultural production from cultivation to sales by managing the soil, water, pesticides, fertilizers, and heavy metals that might remain on the crops. Through the GAP program fertilizers and pesticides were prescribed after analyzing the soil. Soil was sandy clay loam [pH (H_2_O, 1:5): 5.55, total nitrogen: 2.1 g∙kg^−1^, available phosphorus oxide (P_2_O_5_): 278.18 mg∙kg^−1^, exchanged potassium: 51.64 cmoc∙kg^−1^, exchanged calcium: 51.64 cmolc∙kg^−1^, exchanged magnesium: 2.04 cmolc∙kg^−1^].

One of the greenhouses was kept untreated, while the other was treated with pesticide. Each house was divided into three sections that were fertilized with either eco-developed fertilizer (livestock excreta fermentation fertilizer FNC CTCF2; Yooyoo Korea Inc., Jeongeup, Korea), normal fertilizer (functional stevia fertilizer, livestock excreta high-grade fertilizer, Korean stevia; Jeongeup, Korea), or chemical fertilizer (14 ho; Dongbu Farm Hannong, Seoul, Korea) according to the fertilization standards recommended by the National Academy of Agricultural Science, Korea.

### 3.2. Establishment of Fertilizer and Pesticide

The eco-developed fertilizer section was established using eco-developed fertilizer (310 kg/10a) with mixed organic fertilizer (100 kg/10a, Green Soil N-P-K 4-1-3; Biogreentech Inc., Damyang, Korea). The normal fertilizer section was established using 310 kg/10a chemical fertilizer that was processed with chemicals before culturing (urea, 6 kg/10a; phosphatic fertilizer, 3 kg/10a; and potassium chloride, 9 kg/10a). The pesticide used was indoxacarb, which is commonly used as an insecticide. According to the maximum residue limits (MRLs) of pesticides used in Korea, 3.3 g of Indoxacarb was diluted into 20 L of water and applied once for the cultivation periods. Chicory plants were cultivated between 26 April and 30 May in 2014 for 28 days, which corresponds to the spring season. The experimental field design for the six treatments is shown in Table 6.

**Table 6:** Experimental field design.

Groups		Treatment
NP	eco	eco-developed fertilizer without pesticide
	org	organic fertilizer without pesticide
	che	chemical fertilizer without pesticide
LP	eco	eco-developed fertilizer with pesticide
	org	organic fertilizer with pesticide
	che	chemical fertilizer with pesticide

### 3.3. General Composition of Chicory and Reagents

After removal of foreign material in the collected chicory, it was ground using a stainless steel grinder (Hanil, Incheon, Korea) and freeze dried in a plastic bag. The moisture and ash content of the chicory was measured using the AOAC method. Composition analyses were assessed by the Dasan Korea Food Research Institute, Seongnam, Korea. The following methods were used: proteins by the Kjeldahl method (Nitrogen factor: 6.25), fiber by food ingredient test methods of the Korean Food Standards Codex (KFSC, 2013), minerals (Ca, Fe, Mg, and K) by ICP-AES methods. The general components of chicory in the six groups are presented in Table 7.

**Table 7:** Proximate composition and mineral content of chicory.

	NP			LP			Unit
	eco	org	che	eco	org	che	
Protein	20.2	22.6	21.3	14.6	17.1	18.2	g/100 g
Fiber	36.1	36.2	37.0	40.3	37.5	39.7	g/100 g
Ca	1042.5	1010.1	1003.4	1138.5	789.6	889.5	mg/100 g
Fe	29.9	29.5	24.6	20.8	53.1	58.4	mg/100 g
Mg	289.0	332.6	259.6	196.5	236.3	287.6	mg/100 g
K	4850.5	4474.8	5846.8	6402.7	5109.6	4942.3	mg/100 g

NP: non-pesticide group; LP: low-pesticide group; eco: eco-developed fertilizer; org: organic fertilizer; che: chemical fertilizer.

### 3.4. Sample Extraction

Freeze dried chicory powder (10 g) was extracted on an orbital shaker set at 120 rpm for 8 h at 37 °C with 250 mL of 80% ethanol. The supernatant of the mixture was collected in a beaker and pellets were re-extracted under identical conditions two more times. The collected supernatants were filtered using Whatman filter paper (Whatman™ No.1; Whatman International Ltd., Maidstone, England) and concentrated using a rotary vacuum evaporator (N-1110; Eyela, Tokyo, Japan) followed by freeze drying. The freeze dried samples were then stored at −20 °C until analysis. The final samples were diluted according to the requirements of the assays. 

### 3.5. Phenolic Compounds

#### 3.5.1. Total Polyphenol Assay

Total polyphenol content was determined using Folin-Ciocalteu’s reagent and the method of Velioglu *et al.* [37]. Briefly, 2% sodium carbonate (2 mL, Sigma-Aldrich Co., St. Louis, MO, USA) was added to 100 μL of extract. After incubating for 3 min at room temperature, 50% (*v*/*v*) Folin-Ciocalteu’s reagent (100 μL, Sigma-Aldrich Co.) was added. The mixture was incubated for 30 min at room temperature (RT), and the absorbance was measured at 750 nm using a UV-visible spectrophotometer (Shimadzu, Kyoto, Japan). The results are expressed as mg of gallic acid (Sigma-Aldrich Co.) equivalent per g of dry weight (mg·GAE/g).

#### 3.5.2. Total Flavonoid Assay

Total flavonoid content in chicory was measured by using the method of Jia and Wo with slight modifications [33]. The extracts (25 μL) were added to 5% sodium nitrite (8 μL, Sigma-Aldrich Co.) and after 5 min of incubation at RT, 15 μL of 10% aluminum chloride hexahydrate (Duchefa, Haarlem, The Netherlands) was added. After incubation for 6 min at RT, 1 M sodium hydroxide (50 μL, Samchun Chemicals, Phyeontaek, Korea) was added and thoroughly mixed, and finally distilled water (27 μL) was added. The absorbance was measured at 530 nm using a microplate reader (MRX II; Dynex Technologies, Chantilly, VA, USA). Quercetin (Sigma-Aldrich Co.) was used as standard and the content of total flavonoids is represented as mg of quercetin equivalents (mg·QE/g).

### 3.6. Antioxidant Activity

#### 3.6.1. Reducing Power

The reducing power of the chicory extracts (concentration: 1 mg∙mL^−1^) was determined by the method of Mau *et al.* [46]. Chicory extracts (250 μL) were mixed with 0.2 M sodium phosphate buffer (250 µL, pH 6.6) and 1% (*w*/*v*) potassium ferricyanide (Sigma-Aldrich Co.) followed by incubation at 50 °C for 20 min. Then, the extracts were mixed with 1% (*w*/*v*) trichloroacetic acid (250 µL, Sigma-Aldrich Co.) and centrifuged at 10,000 rpm (Micro 17R; Vision Scientific Co., Seoul, Korea) for 10 min. The supernatant (500 μL) was mixed with distilled water (500 µL) and 0.1% (*w*/*v*) ferric chloride (100 µL, Sigma-Aldrich Co.). The reducing power of the samples was detected by measuring absorbance at 700 nm using a spectrophotometer.

#### 3.6.2. DPPH Radical Scavenging Activity.

The scavenging activity of the chicory extracts (concentration: 1 mg∙mL^−1^) was evaluated colorimetrically as described by Hatano [47] with modifications. 1,1-Diphenyl-2-picrylhydrazyl (DPPH, 160 μL, Sigma-Aldrich Co.) was added to the extracts (40 μL). After 30 min, the change in absorbance was measured. With the same concentration of Trolox, DPPH radical scavenging activity was calculated using the following formula:

DPPH radical scavenging activity (%) = (control − sample/control) × 100
(1)

#### 3.6.3. ABTS^+^ Radical Scavenging Activity

Total antioxidant activity of the chicory extracts (concentration: 1 mg∙mL^−1^) was determined using 2,2′-azino-bis-(3-ethylbenzthiazoline-6-sulfonic acid) diammonium salt (ABTS) according to the method described by Re [48]. To generate ABTS anions, 7.4 mM ABTS (Sigma-Aldrich Co.) and 2.6 mM potassium persulfate (Sigma-Aldrich Co.) were mixed and allowed to sit for a day. The ABTS^+^ solution was further diluted in distilled water to give absorbance values between 1.2–1.5 using mol absorbance (ε = 1.6 × 104 M^−1^∙cm^−1^). The chicory extracts (50 μL) were added to diluted ABTS^+^ antioxidants (960 μL), and the change in absorbance was measured after 30 min. Total antioxidant activity was calculated as a percentage (%) using a method identical to that used for a Trolox (Sigma-Aldrich Co.) standard.

### 3.7. Total Carotenoids

After adding hexane (50 mL) to freeze dried chicory powder (0.1 g), the samples were incubated for 1 h at RT. Then, the samples were centrifuged and 100 mL of supernatant was collected. Total carotenoids were measured at an absorbance of 483 nm using a UV-Vis spectrophotometer (V-550; Jasco, Japan) and calculated as follows:

Carotenoids (mg/100 g) = absorbance × vol. × 1000/E^1%^1_mL_ (2592) × sample weight (g) (2)

### 3.8. Vitamins

Metaphosphoric acid (4%, 7 mL) was added to freeze dried chicory powder (200 mg) and after extraction at 4 °C for 2 h, the samples were centrifuged at 9000 × *g* for 10 min (Beckman, Fullerton, CA, USA). The supernatant was collected and filtered with a 0.45 μm syringe filter and used for analysis. Vitamins were separated on a Capcell Pak C18 column (4.6 mm × 250 mm ID, 5 μm) along with mobile phases; 1.25 mM PIC B7 (1-heptanesulfonic acid sodium salt) + 1% acetic acid (*v*/*v*) (solvent A) and 1.25 mM PIC B7 + 1% acetic acid in methanol:water = 60:40 (solvent B) at a flow rate of 1.0 mL∙min^−1^ at 40 °C and a sample injection flow rate of 20 μL. The content of vitamins was quantified using ascorbic acid and folic acid (Sigma-Aldrich Co.) as standards at an absorbance of 280 nm.

### 3.9. Cell Culture and MTT

Human hepatocellular carcinoma cell lines (HepG2) (Korean Cell Line Bank, Seoul, Korea) were initially maintained in Dulbecco’s Modified Eagle’s medium supplemented with 10% fetal bovine serum (FBS; Gibco, Grand Island, NY, USA) and 1% antibiotics (Hycolone, Logan, UT, USA) in a humidified atmosphere of 95% air and 5% CO_2_ at 37 °C. The proliferation of HepG2 cells was measured using the MTT assay. HepG2 cells were resuspended at a final concentration of 1 × 10^4^ and incubated overnight followed by the addition of different chicory samples and further incubation for 24 h. After completion of the incubation period, the spent media were replaced with fresh media containing MTT [3(4,5-dimetyl-2-thiazolyl)-2,5-diphenyl-2*H*-tetrazolium bromide, EZ-Cytox, Daeil Lab Service, Seoul, Korea] and incubated for 4 h followed by measurement of the absorbance at 450 nm using a microplate reader (MRX II, Dynex Technologies, Chantilly, VA, USA).

### 3.10. Statistics

All values are expressed as means ± standard deviation. In order to determine the effects of pesticide, fertilizer, and the interaction between pesticide and fertilizer, two-way factorial ANOVA was performed. Significant differences among values were determined by performing analysis of variance (ANOVA) and Duncan’s multiple range test (MRT). Statistical significance was set at the *p* < 0.05 confidence level.

## 4. Conclusions

To summarize, this study focused on quantifying the antioxidant content of *Cichorium intybus* L., a plant generally enjoyed worldwide. There is still very limited information on its cultivation conditions with respect to enhancing its antioxidant capacity. The results of the experiments performed in this study proved that the type of fertilizer and pesticide did significantly affect the antioxidant properties of *Cichorium intybus* L. It was observed bigger plant size in the pesticide group along with vitamin C content. In addition to this, specifically, antioxidants including total polyphenols and flavonoids along with their antioxidant capacity measured as reducing power and DPPH radical scavenging activity were higher in chicory samples treated with chemical fertilizer and no pesticide and also higher in chicory samples treated with eco-developed fertilizer and pesticide. Total carotenoids and the HepG2 cells inhibitory effect were highest in the eco-developed fertilizer group under both conditions. Therefore, from this study, it can be concluded that eco-developed fertilizer shows more beneficiary effects compared to chemical fertilizers; however, more studies are required to explain why the antioxidant and antioxidant capacity was low for eco-developed fertilizer without pesticides. Finally, we demonstrated not only single effects of pesticides or fertilizers but also multiple effects on chicory.

## References

[1] Søltoft M., Nielsen J., Holst Laursen K., Husted S., Halekoh U., Knuthsen P. (2010). Effects of organic and conventional growth systems on the content of flavonoids in onions and phenolic acids in carrots and potatoes. J. Agric. Food Chem..

[2] Bernard S., Christopher P. (2014). World Cancer Report 2014.

[3] Zhang Y., Han L., Ye Z., Li H. (2014). Ascorbic acid accumulation is transcriptionally modulated in high-pigment-1 tomato fruit. Plant Mol. Biol. Rep..

[4] Health Expenditure, Total (% of gdp). http://data.worldbank.org/indicator/SH.XPD.TOTL.ZS.

[5] Waterland R.A., Jirtle R.L. (2003). Transposable elements: Targets for early nutritional effects on epigenetic gene regulation. Mol. Cell. Biol..

[6] Ornish D., Weidner G., Fair W.R., Marlin R., Pettengill E.B., Raisin C.J., Dunn-Emke S., Crutchfield L., Jacobs F.N., Barnard R.J. (2005). Intensive lifestyle changes may affect the progression of prostate cancer. J. Urol..

[7] Gardener H., Wright C.B., Gu Y., Demmer R.T., Boden-Albala B., Elkind M.S., Sacco R.L., Scarmeas N. (2011). Mediterranean-style diet and risk of ischemic stroke, myocardial infarction, and vascular death: The northern manhattan study. Am. J. Clin. Nutr..

[8] Kontou N., Psaltopoulou T., Panagiotakos D., Dimopoulos M.A., Linos A. (2011). The mediterranean diet in cancer prevention: A review. J. Med. Food.

[9] Gross-Steinmeyer K., Stapleton P., Liu F., Tracy J., Bammler T., Quigley S., Farin F., Buhler D., Safe S., Strom S. (2004). Phytochemical-induced changes in gene expression of carcinogen-metabolizing enzymes in cultured human primary hepatocytes. Xenobiotica.

[10] Block G., Patterson B., Subar A. (1992). Fruit, vegetables, and cancer prevention: A review of the epidemiological evidence. Nutr. Cancer.

[11] Giampieri F., Alvarez-Suarez J.M., Battino M. (2014). Strawberry and human health: Effects beyond antioxidant activity. J. Agric. Food Chem..

[12] Omar N.F., Hassan S.A., Yusoff U.K., Abdullah N.A.P., Wahab P.E.M., Sinniah U.R. (2012). Phenolics, flavonoids, antioxidant activity and cyanogenic glycosides of organic and mineral-base fertilized cassava tubers. Molecules.

[13] Watanabe M., Ohta Y., Licang S., Motoyama N., Kikuchi J. (2015). Profiling contents of water-soluble metabolites and mineral nutrients to evaluate the effects of pesticides and organic and chemical fertilizers on tomato fruit quality. Food Chem..

[14] Esther G., Newark N.J. Dirt Poor: Have Fruits and Vegetables become Less Nutritious?. http://www.scientificamerican.com/article/soil-depletion-and-nutrition-loss/.

[15] Sinkovič L., Demšar L., Žnidarčič D., Vidrih R., Hribar J., Treutter D. (2015). Phenolic profiles in leaves of chicory cultivars (*Cichorium intybus* L.) as influenced by organic and mineral fertilizers. Food Chem..

[16] Biesiada A., Kołota E. (2010). The effect of nitrogen fertilization on yielding and chemical composition of radicchio chicory for autumn-harvest cultivation. Acta Sci. Pol. Hortorum Cultus.

[17] Bunea C.-I., Pop N., Babeş A.C., Matea C., Dulf F.V., Bunea A. (2012). Carotenoids, total polyphenols and antioxidant activity of grapes (*Vitis vinifera*) cultivated in organic and conventional systems. Chem. Cent. J..

[18] Tlili N., Mejri H., Yahia Y., Saadaoui E., Rejeb S., Khaldi A., Nasri N. (2014). Phytochemicals and antioxidant activities of *Rhus tripartitum* (Ucria) fruits depending on locality and different stages of maturity. Food Chem..

[19] Francke A., Majkowska-Gadomska J. (2008). Effect of planting date and method on the chemical composition of radicchio heads. J. Elementol..

[20] Street R.A., Sidana J., Prinsloo G. (2013). *Cichorium intybus*: Traditional uses, phytochemistry, pharmacology, and toxicology. Evid. Based Complement. Alternat. Med..

[21] Heimler D., Isolani L., Vignolini P., Romani A. (2009). Polyphenol content and antiradical activity of *Cichorium intybus* L. from biodynamic and conventional farming. Food Chem..

[22] Rozpądek P., Wężowicz K., Stojakowska A., Malarz J., Surówka E., Anielska T., Ważny R., Miszalski Z., Turnau K. (2014). Mycorrhizal fungi modulate phytochemical production and antioxidant activity of *Cichorium intybus* L. (*Asteraceae*) under metal toxicity. Chemosphere.

[23] Shad M., Nawaz H., Rehman T., Ikram N. (2013). Determination of some biochemicals, phytochemicals and antioxidant properties of different parts of *Cichorium intybus* L.: A comparative study. J. Anim. Plant Sci..

[24] Orhan I.E., Senol F.S., Ozturk N., Celik S.A., Pulur A., Kan Y. (2013). Phytochemical contents and enzyme inhibitory and antioxidant properties of *Anethum graveolens* L.(dill) samples cultivated under organic and conventional agricultural conditions. Food Chem. Toxicol..

[25] Vinha A.F., Barreira S.V., Costa A.S., Alves R.C., Oliveira M.B. (2014). Organic versus conventional tomatoes: Influence on physicochemical parameters, bioactive compounds and sensorial attributes. Food Chem. Toxicol..

[26] Pie J.E., Jang M.S. (1995). Effect of preparation methods on yulmoo kimchi fermentation. J. Korean. Soc. Food Nutr..

[27] Woese K., Lange D., Boess C., Bögl K.W. (1997). A comparison of organically and conventionally grown foods—Results of a review of the relevant literature. J. Sci. Food Agric..

[28] Shier N.W., Kelman J., Dunson J.W. (1984). A comparison of crude protein, moisture, ash and crop yield between organic and conventionally grown wheat. Nutr. Rep. Int..

[29] Lambeth J.D. (2007). Nox enzymes, ros, and chronic disease: An example of antagonistic pleiotropy. Free Radic. Biol. Med..

[30] Rodrigo R., Libuy M., Feliu F., Hasson D. (2014). Polyphenols in disease: From diet to supplements. Curr. Pharm. Biotechnol..

[31] Kähkönen M.P., Hopia A.I., Vuorela H.J., Rauha J.P., Pihlaja K., Kujala T.S., Heinonen M. (1999). Antioxidant activity of plant extracts containing phenolic compounds. J. Agric. Food Chem..

[32] Silva M.M., Santos M.R., Caroço G., Rocha R., Justino G., Mira L. (2002). Structure-antioxidant activity relationships of flavonoids: A re-examination. Free Radic. Res..

[33] Zhishen J., Mengcheng T., Jianming W. (1999). The determination of flavonoid contents in mulberry and their scavenging effects on superoxide radicals. Food Chem..

[34] Ibrahim M.H., Jaafar H.Z., Karimi E., Ghasemzadeh A. (2013). Impact of organic and inorganic fertilizers application on the phytochemical and antioxidant activity of Kacip Fatimah (*Labisia pumila* Benth). Molecules.

[35] Siddiqui Z.S., Ahmed S. (2006). Combined effects of pesticide on growth and nutritive composition of soybean plants. Pak. J. Bot..

[36] Mathew S., Abraham T.E., Zakaria Z.A. (2015). Reactivity of phenolic compounds towards free radicals under *in vitro* conditions. J. Food Sci. Technol..

[37] Velioglu Y., Mazza G., Gao L., Oomah B. (1998). Antioxidant activity and total phenolics in selected fruits, vegetables, and grain products. J. Agric. Food Chem..

[38] Gugała M., Zarzecka K. (2012). Vitamin C content in potato tubers as influenced by insecticide application. Pol. J. Environ. Stud..

[39] Rashida E., El Fadil E.B., El Tinay A.H. (1997). Changes in chemical composition of guava fruits during development and ripening. Food Chem..

[40] Esteban R.M., Molla E.M., Robredo L.M., Lopez-Andreu F.J. (1992). Changes in the chemical composition of eggplant fruits during development and ripening. J. Agric. Food Chem..

[41] Bajaj K., Mahajan R. (1977). Influence of some nematicides on the chemical composition of tomato fruits. Qual. Plant..

[42] Fidalgo F., Santos I., Salema R. (2000). Nutritional value of potato tubers from field grown plants treated with deltamethrin. Potato Res..

[43] Marwaha R. (1988). Nematicides induced changes in the chemical constituents of potato tubers. Plant Foods Hum. Nutr..

[44] Tanaka T., Shnimizu M., Moriwaki H. (2012). Cancer chemoprevention by carotenoids. Molecules.

[45] Liu R.H. (2003). Health benefits of fruit and vegetables are from additive and synergistic combinations of phytochemicals. Am. J. Clin. Nutr..

[46] Mau J.L., Lin H.C., Song S.F. (2002). Antioxidant properties of several specialty mushrooms. Food Res. Int..

[47] Hatano T., Edamatsu R., Hiramatsu M., Mori A., Fujita Y., Yasuhara T., Yoshida T., Okuda T. (1989). Effects of the interaction of tannins with co-existing substances. Vi: Effects of tannins and related polyphenols on superoxide anion radical, and on 1,1-diphenyl-2-picrylhydrazyl radical. Chem. Pharm. Bull..

[48] Re R., Pellegrini N., Proteggente A., Pannala A., Yang M., Rice-Evans C. (1999). Antioxidant activity applying an improved abts radical cation decolorization assay. Free Radic. Biol. Med..

